# Nitrous Oxide

**Published:** 1887-05

**Authors:** A. M. Long


### Nitrous Oxide.

BY A. M. LONG.

(Paper read before the Michigan State Dental Association March 24th, 1887.)

In entering upon the subject of nitrous oxide it is not my purpose to go into its discovery, early history, the process of its manufacture, or means by which its purity may be determined; neither shall I enter upon its physiological action, or whether it enters in combination with the blood and is there decomposed, or whether it is eliminated from the lungs as nitrous oxide without exerting any chemical influence on the blood. I shall confine myself more to the method and manner of its administration. In the year 1844 by mere accident Dr. Wells discovered that this agent could be made to serve a practical purpose; but unlike many in our profession who prefer trying all new experiments on other people, we are informed that he himself inhaled the gas and had a large molar extracted. Many experiments followed with credit and discredit, but it was not so generally known until Colton opened a dental office in New York in 1863. This success with nitrous oxide was soon heralded from the Atlantic to the Pacific, and across the waters to all parts of the civilized world.

Who does not remember the great glad hope that was associated at this time with the word nitrous oxide, but we were buoyant only for a brief time, for the lurking poison in it sent it almost beyond our grip as an anæsthetic. How with bated breath we read of its baleful influence, spasms of the larynx, spasms of the respiratory muscles, continuing for days and sometimes for weeks; vomiting combined with syncope; a deep sleepy condition, as if the patient had taken a powerful narcotic. Headache often severe and lasting from one to four days. Strange sensations or absence of ordinary sensations. In some cases the asphyxiated appearance was strangely marked, the face becoming black, or of a dark purple color. Sometimes the breathing was shallow, and rapid, and puffing, very common in nervous females, and occurring soon after the inhalation had commenced. This accompanied often with screams and nervous twitching. A little later in the administration we find the patient resisting respiration, in which case the breath may be held five or six seconds, and then again the patient may be gasping as for want of air, and the appearance decidedly alarming. Allow me to suggest here if any of these symptoms should present themselves, the dentist should examine the gas so as to be sure of no impurities, and he should thoroughly examine the apparatus and the inhaler to see that every thing is in perfect working order, that no air leaks through and into an imperfect hose or tubing, and that the inhaler valves are free and in perfect working order. The question naturally arises, what has been the cause of the above mentioned symptoms? I will not attempt to explain in detail all the reasons, but allow me to present what may possibly be the chief cause of a few. The manufacturing of nitrous oxide was then crude compared to the present method, and the administration was then made without thought or skill. It is like every thing else from which we hope for success. First the laws which govern them must be understood. By the slow process of improvement the deadly poisons have been eliminated, as the liquifying of nitrous oxide is a safeguard against nitric oxide which requires a much higher pressure to liquify than nitrous oxide.

Next to pure gas, a perfect working apparatus and the inhaler, are essential to the successful administration. The tube on the gas apparatus and the inhaler must be large enough to admit of a free flow of gas, so that nervous patients as well as those with weak lungs can breathe through it without the least exertion. In the construction of the inhaler a few primary and essential principles must not be overlooked. The diameter of the inhaler must be large enough for the passage of the breath as freely as if no covering was over the nose and mouth. The valves must be light and without hinge or spring so that the slightest pressure from exhaling and inhaling will open and close them. The valves opening must be large so that they do not in the least possible degree retard respiration. This is more important with the exhaling, than the inhaling valve. Bear in mind that ordinarily the expiration is made in less time than the inspiration, and it will not be difficult to realize that the opening of the exit valve should be larger than that of the inhalent. Then again the gas receiver can be so constructed as to force the gas through the inhaling valve and naturally aid the patient, but this cannot be done with the exhaling valve. And right here it may be well to say that there must not be allowed in any way any obstruction in the gas channel and it should be large without break or curve. The valves should be close to the mouth, so as to receive full pressure from respiration.

Few people can realize how easily the respiration is impaired. Persons suffering from asthma, angina pectoris, and other conditions, claim that if they sit within two feet, facing a wall, that it causes a strong feeling of suffocation. This I mention to impress upon the profession how important it is that we give attention to the expiration, and especially while we are dealing with an agent that is lighter than air.

The gas receiver must be so sensitive as to respond to the least possible respiration, and it must continue throughout the entire operation. There must be no hindrance whatever, and especially when the patient becomes partly stupified, the least hitch or any abnormal labor of the lungs will stop them then and there and the effect will be incomplete. The patient must not be crowded in the least, nor must he be expected to labor in obtaining it. The receiver should stop with the least pressure of the breath, and start its downward march, at each inhalation, without effort from the patient.

The receiver should supply gas to the patient by forcing it through the long hose into the inhaler, and there be ready for the patient to receive it. By using such an apparatus as I have just described the operator may feel that half the battle is won : then by using pure gas and following the rules that I shall endeavor to give the operator need not expect to see in his patients lividity of complexion and have them drift into dreams of the most frightful character, and become unmanageable in their excitement, neither may he expect to see many other alarming symptoms that arise and are so generally spoken of in the journals and text books. It has been thoroughly proven that nitrous oxide will not support life, yet I am led to believe that fully two-thirds of the alarming symptoms that have been recorded can be directly traced to improper administration.

I will here give an extract showing how one of the professors in the Ohio Dental College was impressed the first time he witnessed the administration of nitrous oxide. He said to an associate " I have been unfavorably impressed with nitrous oxide and learning that you are pleased with it, I have concluded to get more light." An engagement was made and he brought a delicate lady patient to have several teeth removed. The success was all that could be desired, and after the operation, said he: " I took my little boy to a nitrons oxide office and he inhaled the gas from a rubber bag and became very dark in the face, as dark as if he had an epileptic seizure. I did not like it." Many dentists who are not familiar with gas imagine that it is too transient to last longer than to extract one tooth, and it is to intensify the natural disposition of the person who takes it. A man of pugnacity will become more belligerant for the moment. Hilarious persons are exceedingly jolly. Nervous people are apprehensive of danger, and cannot be brought under its influence at all, and for the above reasons if an anæsthetic is required they prefer chloroform or ether. The frequent deaths caused by chloroform and ether as anæsthetics have often been impressed upon us. Should we, as a profession, having the entire control of our confiding patients who place themselves in our care for treatment, use so deadly and so treacherous an agent as chloroform for extracting teeth? No security against its fatal tendency is known. No reliable rules by which to select a subject exempt from its deadly influence; no antidote sufficient, or means of resuscitation deserving of much confidence.

Who does not feel this professional responsibility to a painful degree, so as not to ignore the value of the lives of his patrons, and heedlessly disregard the inteligent warnings of statistical records, may some day wish he had never entered upon dentistry or surgery. Some dental practitioners condem the use of anæsthetics in dental practice and will not use them at all, claiming for their argument that it divests the operation of all its terrors, and therefore causes persons to have teeth extracted that should not be. This alludes to wholesale extracting institutions, that have opened offices all over our broad land, and are sacrificing good teeth by the thousands. Victimizing their patients with rose-tinted, perfumed, nitrous oxide, with all the arts of handicraft, witchcraft, and other crafts, like the lightning tooth-extractor that sails in upon us with his Maye's vapor, Vitalized air, oxygenated air, medicated vapor, suflogisticated air, etc., at ten cents a ha! ha!

One would naturally believe that this was a merchandise article, and could be held and administered in every household as freely as a bottle of Dr. Bull's Cough Syrup. It is the abuse of nitrous oxide that disgraces our calling, and not its use. For it is one of the grandest gifts to suffering humanity, and as yet, nothing has ever been discovered offering the safety and advantages which this gas possesses, although it has not proven to be absolutely free from danger.

When I can take a man or woman, old or young, who comes to my office, free from liquor and is self-possessed and composed, and confident that they are not going to be hurt; when I have these patients sitting in my chair, and take the gas without any struggle, and have their teeth extracted without a particle of pain, and when I see them within ten minutes go about their business as though nothing had happened, when I see this day after day, year in and year out, I can not refrain from the belief that nitrous oxide has a legitimate place in dentistry.

The question which may occupy our attention is whether there are any known conditions of the system in which nitrous oxide should not be given. Patients who suffer from acute rheumatism and heart trouble, such as fat heart or damaged valves, epilepsy, St. Vitus' Dance, disease of the brain, lungs, and kidneys, are not desirable patients for the administration of anæsthetics.

Our experience informs us that it is no barrier to this agent, if a plentiful supply be given, and the patient is not asphyxiated long before the gas has time to produce anæsthesia.

This is a common occurrence when giving gas from the bag, and the long hose that has been attached for an improvement over holding it in the lap, for it requires force to draw air or gas through a long tube.

If there should be fluttering of the pulse, or irregularity, or running faster than normal for the age of the patient, the color of the face should indicate low temperature, and the patient appear to be low in vitality.

Under these manifestations of abnormality nitrous oxide may, in many instances, be administered with comparative safety, while the indications show that neither ether or chloroform should be given.

Here are an unlimited range of subjects presenting themselves for our consideration. The youth with his vigorous and strong circulation, even in health, is different from the adult, with his steady-flowing arterial rivers; and a still greater difference in the senile man, sinking the flood of his once active heart into the sands of old age; say nothing of the mental conditions of different subjects, and at different times. Look you, even in health, that you deal not with these classes all alike. The youth may hold his breath for two or three seconds with no alarming symptoms, yet in old age if this should go on unnoticed for a few seconds it may be the closing scene of a precious life.

The operator should allow nothing to distract his attention from his patient while administering an anæsthetic, and if any alarming symptoms should occur, such as stopping of breathing, or loss of pulse, the patient should have immediate attention. The operator must not lose his own balance, but proceed with all known means of restoration. If the breathing ceases he should thrust his forefinger low down into the throat, and draw the tongue forward and hold it. This will exact some motion of the throat and mouth, and may be that is all that is necessary. If this should fail then slapping the chest with the hand, or holding a wet cloth to the face, applying ammonia to the nostrils, or the vapor of amyl nitrate, elevating the limbs and rubbing the extremities towards the bodies; also raise the feet and lower part of the body higher than the head. Expand the chest by pressing the sides, and thus induce breathing if possible. Apply the battery and work vigorously, for moments of time are precious.

Before closing, allow me to state in brief what may yet seem essential to success. First get the full confidence of the patient. The patient should be as much as possible in a horrizontal position, for these reasons: the heart, being the medium of force in the circulation of the blood, and as it is located near the center of the body, and the laws of gravitation hold good with the course of blood from the arteries in the upper portion of the body and the veins in the lower portion, hence we must recline the body to equalize the force, to overcome gravitation, and assist the diastolic action of the heart when it becomes weakened by anæsthesia. Instruct the patient to loosen the clothing, especially around the neck and waist, and avoid tight fitting shoes and gloves, and in fact lessen every burden of resistance to the physical force. Place a large rubber apron around the neck and over the lap of the patient, to prevent the clothing from being soiled, then by kind and cheerful words allay fear and gain the confidence of the patient in your ability to administer an anæsthetic.

Allow the patient a small quantity of water, so as to moisten the mouth and throat, as it is in its normal condition, for the salivary glands are apt to be inactive while the patient is laboring under the excitement of an operation.

Examine the teeth, and place the jaw or face opposite the teeth to be extracted. An assistant should stand at the chair ready to hand forceps and help in case it is needed. See that every thing is ready before operating, as a few seconds occupied in finding an instrument may result in failure. Every thing being ready, instruct the patient to take five or six inhalations, and as rapid as possible, say five times as fast as possible, keeping at this time the inhaler away from the face. This full and rapid breathing familiarizes the patient, and gets him accustomed to fill the lungs; but the main object is to change residual air in the air cells of the lungs. While the lungs are empty place the inhaler over the mouth and nose, and allow the patient to take two or three inhalations of nitrous oxide, after which remove the inhaler, and allow the patient to take about the same amount of air before replacing the inhaler. The object is to accustom the air cells in the lungs to the gas gradually. From this on throughout the operation the patient should be instructed to breathe as naturally as possible.

As soon as the operation is performed the nostrils should be moistened with aqua ammonia. This will not only stimulate the nerve of the nasal membrane, but it will neutralize the carbonic acid in the air cells of the lungs. The vapor of the amyl nitrate is also strongly recommended. The patient should not be kept in a state of semi-consciousness longer than can be helped, and even if they have so far recovered as to be able to speak a small amount of the vapor of ammonia will be beneficial.

Between the stages of reasoning and complete anæsthesia the apprehensions of danger from gas and the dread of pain may at this critical moment produce a nervous shock, if a tooth is to be extracted; therefore it can be easily understood why the operation should stop at this point, and revive the patient to full consciousness. A bowl and sponge should be in reach, and as soon as the teeth are extracted the head of the patient should be inclined forward, to prevent the blood from running down the throat and allow it to run into the bowl.

Lady patients should be waited on by a lady assistant after an operation. A third person should always be present with any anæsthetic.

There is but one more thought, then I shall close. The mixing of fluid with nitrous oxide has been strongly condemned by professional men, both in this country and in Europe. The Clover apparatus for administering ether and nitrous oxide gained only a limited reputation, and soon went into disuse, as far as I can learn of its history. Here the operator relies on the ether to produce anæsthesia, using the gas to hasten the narcotizing effects. From my personal observation I can not speak in favor of ether, allowing the quantity to be much or little. Its effects are too irritating. But I have found from six to nine drops of the best of alcohol and chloroform in equal parts, thoroughly vaporized in ten gallons of gas, to be a wonderful improvement in prolonging anæsthesia, and yet while it prolongs anæsthesia it does not change the effects of nitrous oxide, and I have as yet never seen any bad results.

My desire and effort has been to present a clear and concise statement of the manner and method of administering nitrous oxide, and point out that this can only be assured by an intelligent guard that its exhibition be suspended while yet the centers governing respiration and circulation are not two profoundly impressed. I have indicated the responsibility, and I hope what I have said may at least stimulate and put into the hearts of some a desire to gain a better knowledge of nitrous oxide.

---

SHE SWALLOWED HER FALSE TEETH.—A dispatch from Paterson, New Jersey, says that, on December 3, Mrs. Henry Lowe, while drinking a glass of water, loosened a gutta percha plate with five false teeth, and they slipped down her throat. The physician, in trying to get the plate out of the throat, moved it so that it slipped down into her stomach. It is now proposed to remove the teeth by the operation of opening the stomach.—*Med. and Surg. Rep.*

